# Enhancing Cotton Fabrics Through Grafting of Glycine-Based Polyamidoamine

**DOI:** 10.3390/polym17121676

**Published:** 2025-06-17

**Authors:** Matteo Arioli, Jenny Alongi, Claudia Forte, Silvia Pizzanelli, Elisabetta Ranucci

**Affiliations:** 1 Dipartimento di Chimica, Università degli Studi di Milano, via C. Golgi 19, 20133 Milano, Italy; matteo.arioli@unimi.it (M.A.); jenny.alongi@unimi.it (J.A.); 2 Institute of the Chemistry of OrganoMetallic Compounds (ICCOM), National Research Council, via G. Moruzzi 1, 56124 Pisa, Italy; claudia.forte@pi.iccom.cnr.it (C.F.); silvia.pizzanelli@pi.iccom.cnr.it (S.P.)

**Keywords:** polyamidoamine, cotton, durable coating, flame retardancy, photostabilization

## Abstract

Durable polyamidoamine (PAA) coatings were covalently grafted onto cotton by applying a water-soluble, glycine-based PAA (M-GLY) through a radical polymerization mechanism. M-GLY oligomers of different chain lengths, terminated with bisacrylamide groups, were synthesized via polyaddition of N,N′-methylenebisacrylamide and glycine at molar ratios of 1:0.9, 1:0.85, and 1:0.8. Cotton strips were then impregnated with differently concentrated (10 and 20 wt.%) aqueous solutions of the M-GLY oligomers in the presence of potassium persulfate, which oxidized cellulose and generated radicals that initiated polymerization of the M-GLY terminals, thereby enabling covalent grafting onto cotton. This process yielded M-GLY-grafted cotton (COT-g-M-GLY) with 2–15% add-on levels. Scanning electron microscopy revealed uniform surface coverage and penetration of the coating into fiber interiors. Grafting did not alter cellulose crystallinity—65% vs. 64% for grafted and virgin cotton. However, thermogravimetric analysis showed that COT-g-M-GLY exhibited lower thermo-oxidative stability than M-GLY-adsorbed cotton (COT/M-GLY) at similar add-ons. Flame-retardancy tests indicated that COT-g-M-GLY reduced the burning rate (by 10% to 30%) but did not achieve self-extinguishing behavior, unlike COT/M-GLY. Despite this, COT-g-M-GLY provided good protection against UV-induced photodegradation. After accelerated UVA–UVB exposure, cotton samples with 10% M-GLY add-on showed a significantly reduced yellowing rate compared to untreated cotton, as confirmed by spectrophotometric analysis.

## 1. Introduction

Cellulose, the most abundant natural polymer, is valued for being lightweight, biodegradable, and renewable [1]. Graft co-polymerization of cellulose and its derivatives is an effective method for modifying their physical and chemical properties, improving functionality and broadening their applications [2,3]. Among various approaches, graft co-polymerization initiated by chemical treatments, such as free radical polymerization with peroxides and redox systems [4,5,6,7,8], controlled radical polymerization [9,10,11], and ring-opening polymerization [12,13], has gained significant attention over the years. Additionally, techniques involving photo-irradiation [14] and high-energy radiation have been widely explored [15].

As with all cellulosic materials, cotton textiles are highly flammable, with a limiting oxygen index (LOI) value of 16–18% [16,17,18]. They can ignite when exposed to heat sources like flames, sparks, or hot surfaces, and several studies suggest that most indoor fires are linked to the spread of cotton fabric fires [19]. This justifies efforts to develop safe, effective flame retardants (FRs) for cotton. For the past 50 years, phosphorylated compounds such as tetrakis(hydroxymethyl) phosphonium salts (Proban^®^) and N-methyloldialkyl phosphonopropionamides (Pyrovatex^®^) have been the primary FRs used for cotton [20]. However, many of these compounds have a substantial environmental impact due to the release of formaldehyde during both manufacturing and use. Additionally, they generate fumes and smoke when activated [21]. Although extensive research has been conducted on new phosphorylated FRs [22], international regulations [23,24] demand more eco-friendly, non-toxic, and effective FRs for cotton.

It is widely acknowledged that the photodegradation of cellulosic materials, triggered by UV radiation and facilitated by the formation of reactive radicals and oxidized groups, leads to material degradation and aging through radical chain reactions [25,26,27]. Even prolonged exposure of cellulosic artifacts to low-intensity UV radiation, such as sunlight, over several decades gradually leads to C-C and C-O-C bond breakages in cellulose, generating radicals that, in the presence of oxygen, form new chromophores that change the spectral absorption band of cellulose light, responsible for yellowing. Strategies to protect and mitigate the photoaging of cellulose include incorporating photostabilizers with different actions, such as UV absorbers, reactive oxygen species (ROS) scavengers, or antioxidants [28]. In all these applications, reducing the leaching of plastic additives, including FRs and photostabilizers, can improve the environmental sustainability and safety of the final products.

Linear polyamidoamines (PAAs) derived from α-amino acids have been shown to act both as intumescent flame retardants [29] and photostabilizers [30] for cotton. These multifunctional polymers are synthesized by the aza-Michael polyaddition of bis-secondary or primary amines, including α-amino acids, with bis-acrylamides [31]. They can be prepared in concentrated aqueous solution, at room temperature, and without the addition of catalysts. PAAs can be synthesized to be biocompatible and biodegradable and, therefore, find use in biotechnological applications, for instance as drug carriers [32] or non-viral vectors for gene therapy [33]. One of the distinctive features of α-amino acid-derived PAAs is their eco-compatibility [34,35,36]. In the form of thin coatings, these PAAs protect cotton fabrics from photo-oxidation [24]. Their performance was attributed to the radical scavenging ability of tert-amine groups present in their repeat units. Moreover, they protect cotton from ignition and flame propagation in horizontal flame spread tests (HFSTs) [37]. The glycine/L-cystine copolymers have been proven to extinguish the flame also in vertical flame spread tests (VFSTs) [38].

PAAs are highly water-soluble and are quickly washed out from cotton during laundering. To expand the application of PAA coatings in enhancing the physicochemical properties of cotton, it is therefore crucial to covalently bond them to the cotton surface, thereby improving their durability through multiple wash cycles. The aim of this research is to present an easy procedure to chemically modify the cotton surface with PAAs to impart it flame retardancy and photostability.

## 2. Materials and Methods

### 2.1. Materials

N,N’-methylenebisacrylamide (MBA, 99%), glycine (GLY, >99%), sodium hydroxide (NaOH, >99%), D_2_O (99.9%), DCl (35% in D_2_O), fuming hydrochloric acid (HCl, ≥37% in water), lithium hydroxide monohydrate (LiOH·H_2_O, ≥98%), potassium persulfate (>99%), and potassium carbonate (99.9%) were supplied by Sigma Aldrich (Milan, Italy). Cotton fabric (COT) with an area density 250 g m^−2^ was purchased from Fratelli Ballesio S.r.l. (Turin, Italy).

### 2.2. Synthesis of M-GLY Oligomers

*M-GLY_0.85_*: N,N′-methylenebisacrylamide (9.34 g, 60 mmol), glycine (3.87 g, 51 mmol) and lithium hydroxide monohydrate (2.19 g, 51 mmol) were suspended in distilled water (25 mL) and maintained under stirring at 50 °C until complete dissolution. The reacting solution was then left in the dark at 25 °C for five days. The resulting viscous solution was acidified to pH 4 with 6 M HCl and used with no further treatment. A 1 mL aliquot was freeze-dried and analyzed by ^1^H-NMR in D_2_O.

The amounts of reagents used in the preparation of all M-GLY-based oligomers are summarized in Table 1.

An M-GLY sample bearing no acrylamide terminals was obtained following the same procedure adopted in the synthesis of the M-GLY oligomers but using a 1:1 MBA/GLY molar ratio.

The chemical structure of the M-GLY oligomers was assessed by ^1^H-NMR, collecting spectra in D_2_O at pH 4.0 and at 25 °C using a Bruker Advance DPX-400 NMR spectrometer (Milan, Italy) operating at 400.13 MHz. Parameters: scan number 32, relaxation delay (d1) 10.0 s, receiver gain automatically measured and set by the instrument.

### 2.3. M-GLY Grafting onto Cotton Fabrics

A strip of cotton fabric (30 mm × 60 mm) was dried at 80 °C, weighed, then placed in a polypropylene pouch (50 mm × 90 mm), and finally impregnated with an M-GLY solution (750 μL) of suitable concentration (10 or 20 wt.%) containing 1 wt.% potassium persulfate using an electronic micropipette. The pouch was sealed with PET strips using a hot press at 150 °C and then kept at 80 °C for 3 h. After this time, the cotton strip was extracted from the sealed pouch and maintained at 50 °C for 30 min. Then, it was first immersed in distilled water at 45 °C, secondly in 0.01 M HCl at room temperature, and finally in water (100 mL, 30 min each) under vigorous stirring. The resulting M-GLY-grafted cotton fabric was dried to constant weight.

The total dry solid add-on (*Add-on*, wt.%) was calculated according to Equation (1).

(1) $$Add-on\%=\frac{W_{f}-W_{i}}{W_{i}}\times 100$$

where *W_i_* is the weight of the dried sample before treatment and *W_f_* the weight of the dried sample after treatment. The concentrations of the M-GLY solutions used in the cotton grafting experiments are shown in Table 3 (Section 3).

M-GLY-grafted cotton fabrics were labeled as follows: COT-g-M-GLY_0.9_ represents a cotton sample grafted with the M-GLY_0.9_ oligomer. For comparison purposes, a set of M-GLY-adsorbed cotton samples was obtained by impregnating cotton with M-GLY water solutions and then drying. These samples were labeled as COT/M-GLY.

### 2.4. Water Uptake

Water uptake tests were performed on virgin cotton and COT-g-M-GLY samples. Cotton strips (30 mm × 60 mm) were first dried over CaCl_2_ for 4 h and then placed in a chamber maintained at 25 °C and 40% relative humidity, obtained using a K_2_CO_3_ saturated solution, for 15 h. The hydrated samples were then weighed. The water uptake was calculated using Equation (2):

(2) $$Water\;uptake\%=\frac{W_{t}-W_{0}}{W_{0}}\times 100$$

where *W*_0_ = weight of the dry cotton strip and *W_t_* = weight of the hydrated cotton strip.

### 2.5. Solid-State Nuclear Magnetic Resonance

Solid-state NMR (SSNMR) spectra were acquired using a Bruker AVANCE NEO NMR spectrometer operating at Larmor frequencies of 500.13 MHz for ^1^H and 125.77 MHz for ^13^C nuclei. The instrument was equipped with a double-channel (H/F–X) 4 mm CP-MAS probe. The ^1^H–^13^C cross-polarization (CP) experiments were performed with high-power ^1^H decoupling, using a contact time of 1 ms, a recycle delay of 2 s, and acquiring 4000 scans. The 90° pulse durations were 4.3 µs for ^1^H and 4.2 µs for ^13^C. Experiments were conducted under magic-angle spinning (MAS) at a spinning rate of 15 kHz and with high-power ^1^H decoupling. The ^13^C chemical shift scale was referenced to the external adamantane signal at 38.48 ppm.

### 2.6. Scanning Electron Microscopy

The surfaces of virgin cotton and COT-g-M-GLY samples (5 mm × 5 mm) were gold-metallized and then analyzed with a Zeiss field-emission scanning electron microscope (FE-SEM), model ZEISS-SIGMA 300 operating at 8.5 mm working distance and 5 kV beam voltage (Zeiss, Ramsey, NJ, USA).

### 2.7. Thermogravimetric Analysis

The thermal stability of virgin cotton and COT-g-M-GLY samples was assessed by thermogravimetric analysis in nitrogen and air from 50 to 800 °C range, at 10 °C min^−1^ heating rate, with a 50 mL min^−1^ gas flow, using a TGA 2 Star System (Mettler-Toledo, Milan, Italy).

### 2.8. Flame Spread Test

Combustion tests were carried out in horizontal configuration (horizontal flame spread test—HFST). HFSTs were conducted on 30 mm × 60 mm cotton strips mounted on a metallic frame and inclined at a 45° angle along their longitudinal axis. A butane flame (20 ± 5 mm in length) was applied to the transverse (short) edge of each sample for 3 s. Each test was performed in triplicate. The parameters recorded included combustion times in the presence of flame and afterglow (s), burning rate (mm s^−1^), and residual mass fraction (RMF, %).

### 2.9. Accelerated Photoaging Test

Photoaging experiments under accelerated conditions were performed within a solar chamber, internally covered with aluminum foil. Strips 20 mm × 50 mm in size of COT/M-GLY and COT-g-M-GLY_0.85_ with 10% M-GLY add-on, and of virgin cotton were placed on a circular stand rotating at a 40-rpm rate and irradiated for a total of 22 h with a 500 W UV lamp (emission wavelength range: 280–400 nm) positioned 580 mm away from the sample holder. A relative humidity of 40% was obtained by placing a saturated K_2_CO_3_ water solution in the chamber. Every 30 min, irradiation was interrupted for 15 min to regulate the temperature, which varied from room temperature to 75–80 °C during the irradiation cycle. Every 60 min, samples were analyzed by FT-IR and spectrophotometric analyses. Tests were performed at least in duplicate. After each irradiation session, samples were stored in the dark.

Spectrophotometric analyses were performed following the ISO11664 standard, using a CM-2300d spectrophotometer (Konica Minolta, Guangzhou, China) [39].

The following CIELAB color space parameters were recorded: L* (sample brightness, ranging from 0 (black) to 100 (white)), a* (green–red axis), and b* (blue–yellow axis) chromaticity coordinates. The color change (ΔE*) was calculated as described by Equation (3):

(3) $${∆E}^{*}=\sqrt{{\left({∆L}^{*}\right)}^{2}+({{∆a}^{*}{)}^{2}+({∆b}^{*})}^{2}}$$

Chromaticity coordinates and ΔE* have no physical units because they describe perceptual attributes rather than directly measurable physical quantities. ΔE* measures color difference on a perceptual scale reflecting human color perception. Thus, a higher ΔE* indicates greater color changes due to aging. Color differences were categorized as follows: not present (ΔE* = 0); imperceptible to the human eye (0 < ΔE* ≤ 1); perceptible under close observation (1 < ΔE* ≤ 2); immediately noticeable (2 < ΔE* ≤ 10); highly distinct (11 < ΔE* ≤ 49); and extremely distinct (ΔE* ≥ 50) [40].

## 3. Results

### 3.1. Synthesis of M-GLY-Grafted Cotton—COT-g-M-GLY

**Scheme 1:**
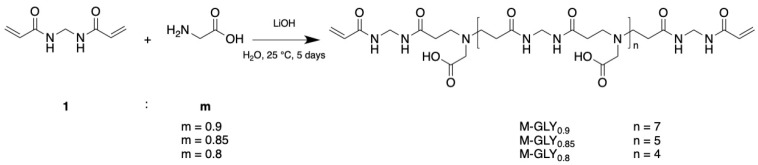
Synthesis of α,ω-acrylamide-terminated M-GLY oligomers.

**Figure 1:**
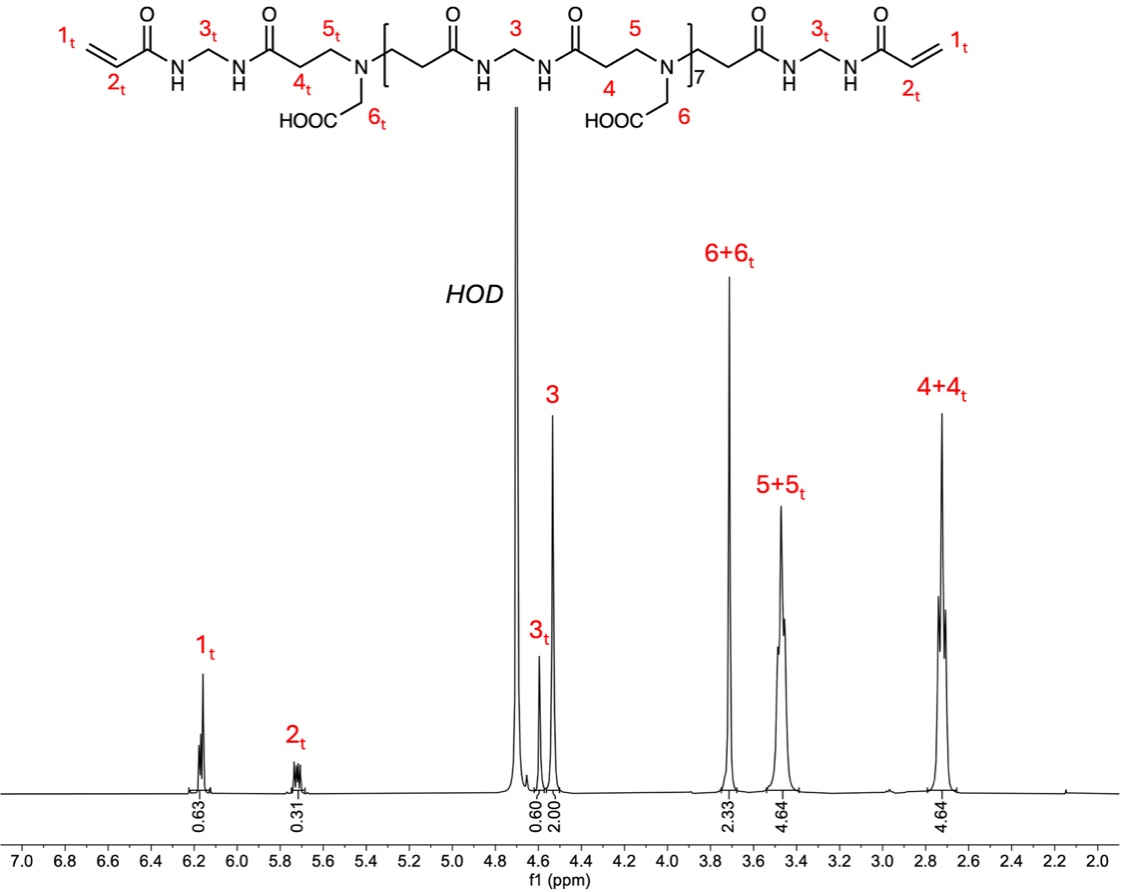
^1^H-NMR spectrum of M-GLY_0.9_ in D_2_O at pH 4.0.

Polyamidoamine oligomers with acrylamide terminals and different polymerization degrees were synthesized through the aza-Michael polyaddition of N,N′-methylenebisacrylamide (MBA) with glycine in 1:0.9, 1:0.85, and 1:0.8 molar ratios (Scheme 1). The resultant oligomers were designated as M-GLY_0.9_, M-GLY_0.85_, and M-GLY_0.80_, respectively, based on the molar ratios of the amine versus acrylamide functions in the monomer mixture. The reaction was conducted under standard experimental conditions, specifically at a 40 wt.% concentration, pH 11, and room temperature [25]. The structure of the M-GLY oligomers was confirmed using ^1^H-NMR spectroscopy (Figure 1 and Figure S1 in the Supplementary Materials), showing agreement with the expected molecular structure. The number-average molecular masses (Table 2) were consistent with theoretical values.

**Scheme 2:**
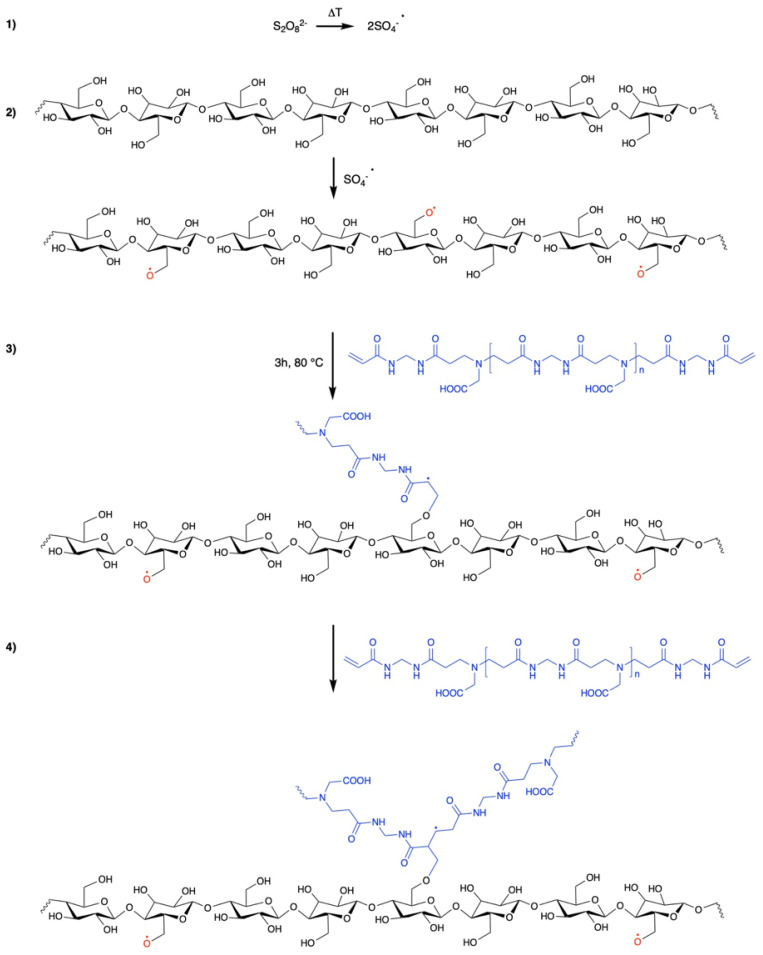
Grafting of α,ω-acrylamide-terminated M-GLY oligomers onto cotton through a radical mechanism.

The grafting of M-GLY oligomers onto cotton proceeded via radical activation of the cotton fibers using potassium persulfate as both an oxidizing agent and polymerization initiator, following a well-established procedure [2]. The process begins with the thermal decomposition of potassium persulfate, generating reactive sulfate radical anions (Scheme 2(1)), which abstract hydrogen atoms from the hydroxyl groups of cellulose, thereby forming O-centered cellulose radicals (Scheme 2(2)). These radicals subsequently react with the terminal acrylamide double bonds of the M-GLY oligomers, initiating radical polymerization (Scheme 2(3,4)). Due to their bifunctional nature, M-GLY oligomers—which can also directly react with sulfate radical anions—generate a crosslinked polymer network. This hypothesis is supported by previous reports on the radical polymerization of α,ω-acrylamide-terminated polyamidoamines [41]. Crosslinking was further confirmed by a control experiment in which an aqueous M-GLY_0.85_ solution, treated with potassium persulfate under the same conditions used for cotton grafting, formed a swollen, crosslinked hydrogel (Figure S2). Notably, although the grafting reactions onto cotton through a radical mechanism are well-known, no previous studies, to our knowledge, have investigated the chemical modification of cotton with polyamidoamines.

Cotton samples grafted with M-GLY (COT-g-M-GLY) were prepared using 10 and 20 wt.% concentrations of the M-GLY_0.90_, M-GLY_0.85_, and M-GLY_0.80_ oligomers. Experiments were conducted at 80 °C with an initiator concentration of 1 wt.% based on the oligomer’s weight. Following thorough washing, the resulting M-GLY add-ons were quantified (Table 3), showing a variation ranging from approximately 2 to 15%. The grafting efficiency was clearly concentration-dependent, with the add-on ranging from 2 to 5.4% at the lowest concentration and from 11 to 15% at the highest. The COT-g-M-GLY samples exhibit slightly higher water uptake compared to plain cotton, which is consistent with the known hydrophilicity of the M-GLY coating. Based on our experience with hydrophilic materials, such measurements typically involve a degree of variability. Therefore, we consider the observed differences in water absorption among the samples to be within experimental uncertainty and not statistically significant.

**Table 3:** Add-on and water uptake ability of COT-g-M-GLY samples. ^(a)^.

	COT-g-M-GLY_0.9_		COT-g-M-GLY_0.85_		COT-g-M-GLY_0.8_	
Solution concentration (wt %)	10	20	10	20	10	20
Add-on (%) ^(b)^	2	11	5.4	11	4.5	15
Water uptake (%) ^(c)^	2.4	2.7	2.7	2.4	2.0	2.4

^(a)^ Reaction conditions: 80 °C, 3 h. ^(b)^ Data represent averages obtained from at least six samples. The experimental error was ± 1%. ^(c)^ Determined at 45% RH and 25 °C for 15 h. The water uptake of virgin cotton under the same conditions was 2.2%. Data represent averages obtained from three samples. The experimental error was ± 1%.

The surface of the COT-g-M-GLY samples was analyzed using FT-IR/ATR spectroscopy (Figure S3a–c). All spectra exhibited broad, intense bands at 3300 cm^−1^, 2960–2830 cm^−1^, and 1100 cm^−1^, corresponding to O–H, C–H, and C–O stretching vibrations of cellulose, respectively. Additionally, weak absorptions in the 1600–1500 cm^−1^ range were attributed to amide N–H bending and C–N stretching from the M-GLY repeat units. Notably, increasing the M-GLY add-on resulted in a slight enhancement of these band intensities. The N–C=O stretching vibration at 1680 cm^−1^ overlapped with the O–H bending band of water.

**Figure 2:**
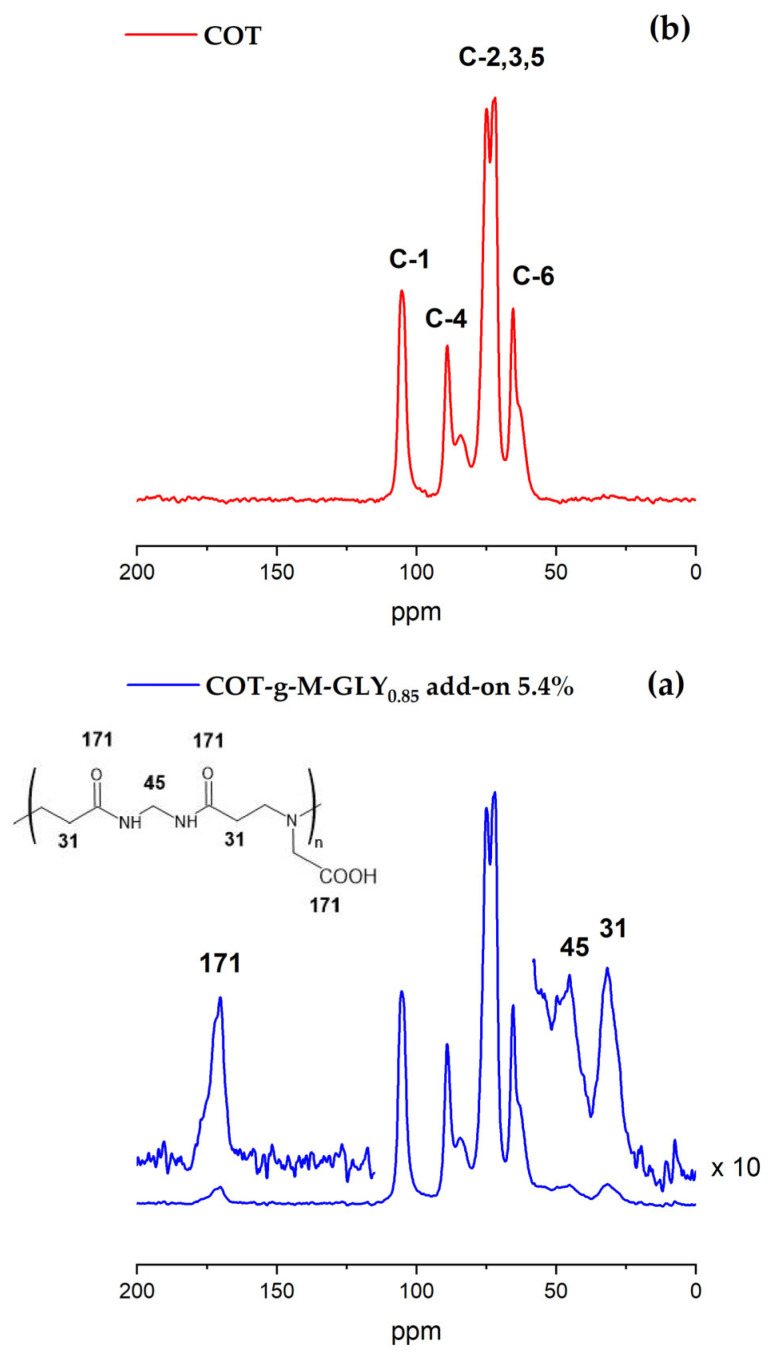
^1^H-^13^C CP-MAS spectrum of COT-g-M-GLY_0.85_ (with a magnification of the M-GLY signals) (**a**) and of pure cotton (**b**).

In Figure 2, the ^1^H–^13^C CP-MAS spectrum of COT-g-M-GLY_0.85_ with a 5.4% add-on and that of pure cotton are compared. The intense signals from cotton appear in the region between 60 and 110 ppm, while the weaker signals, observed between 0 and 60 ppm and at 171 ppm, correspond to carbon atoms in the M-GLY repeat units [42]. The chemical shifts at 31, 45, and 171 ppm are in agreement with those reported for high molecular weight M-GLY in aqueous solution and are attributed to the methylene carbon in the alpha position to the carbonyl group of the amide moiety, the methylene group bonded to two amide nitrogen atoms, and the carbonyl groups of the amide and carboxylic moieties, respectively (see inset in Figure 1). The other carbon atoms of the M-GLY monomer unit are expected to resonate in the 50–60 ppm range, producing a broad signal that partially overlaps with the much more intense signal from cellulose C-6 in cotton. Notably, no signals attributable to terminal alkenyl carbon atoms of the oligomer are observed, suggesting that most, if not all, terminal double bonds have reacted. Based on the data obtained, the grafting site of M-GLY on cotton could not be conclusively identified, as the polymer carbons directly involved in the grafting are expected to give rise to signals that overlap with those of cotton, namely cellulose C-6.

Comparison of the cotton signals in pure cotton and in COT-g-M-GLY_0.85_ reveals no significant differences. In particular, the similarity of the signals of cellulose C-4 and C-6, both resulting from distinguishable contributions from crystalline and amorphous cellulose, indicates that cotton maintains the same crystallinity after reaction with M-GLY. The crystallinity index, determined according to the method of Newman [43], was found to be 54% for both samples (Figure S4). X-ray diffraction (XRD) analysis confirmed that the grafting reaction did not affect the crystallinity of cotton, as demonstrated by the analyses of a COT-g-M-GLY_0.85_ sample with a 5.4% M-GLY add-on and a COT-g-M-GLY_0.8_ sample with 15% add-on. This is evident from the comparison with the XRD pattern of untreated cotton, as shown in Figure S5. Specifically, the XRD spectrum of virgin cotton showed characteristic strong and broad diffraction peaks at 15.21°, 16.76°, and 22.98°, corresponding to the (100), (010), and (110) crystal facets of cellulose (Table S1) [44,45]. In addition, a shoulder around 20° is ascribed to the (012)/(102) facets, while a less intense peak centered at 35.08° corresponds to the (2-1-1)/(210)/(01) crystal facets. No significant shifts were observed in the diffraction peak positions of the two COT-g-M-GLY samples. Similarly, the crystallinity index remained largely unchanged, with values of 65% for COT-g-M-GLY_0.85_ and 66% for COT-g-M-GLY_0.8_, compared to 64% for untreated cotton.

Overall, SS-NMR and XRD analyses yielded different crystallinity values of cellulose, consistent with previous findings in the literature [46]. This variation reflects the well-documented dependence of the crystallinity index on the analytical technique used.

### 3.2. Morphological Analysis

**Figure 3:**
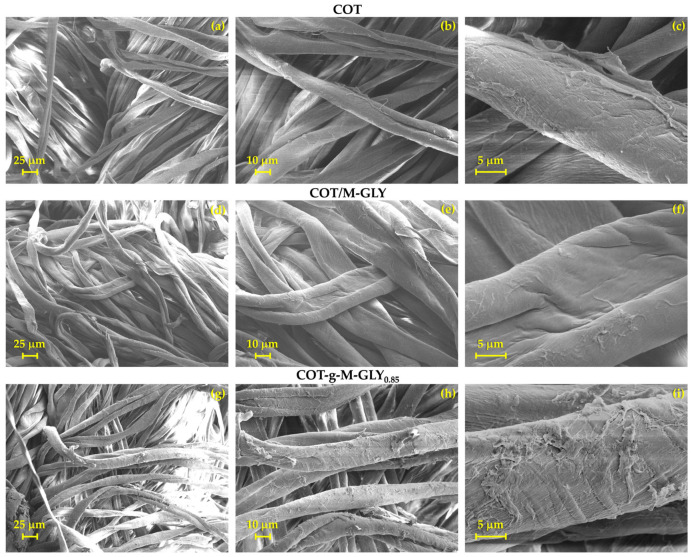
FE-SEM micrographs of COT, COT/M-GLY and COT-g-M-GLY_0.85_ (Add-on: 10%). Magnifications: 1000× (**a**,**d**,**g**), 2500× (**b**,**e**,**h**), 5000× (**c**,**f**,**i**).

The surface morphology of a COT-g-M-GLY_0.85_ sample with a 10% add-on, as a representative example of all prepared samples, was observed by FE-SEM (Figure 3e,f) alongside the images of control virgin cotton (Figure 3a,b) and a COT/M-GLY sample with the same add-on (Figure 3c,d) at the same magnifications. Notably, the effect of COT-g-M-GLY_85_ presence is evident on the surface of the cotton fibers, as indicated by a slight increase in surface roughness observed at the microscopic scale. However, in both COT/M-GLY and COT-g-M-GLY_0.85_, the natural spiral structure of the cellulose fibers was preserved. The fibers maintained their individuality and exhibited flat, smooth surfaces, indicating that the grafting process did not significantly alter the original morphology of the fabric. Additionally, a continuous and uniform coating was evident on both the surface and within the interior of the cotton fibers. At higher magnification, some regions of COT-g-M-GLY_0.85_ exhibited lower uniformity and homogeneity than COT/M-GLY (Figure 3f vs. Figure 3d), possibly due to structural rigidity introduced by the grafting procedure.

### 3.3. Thermal Analysis

**Figure 4:**
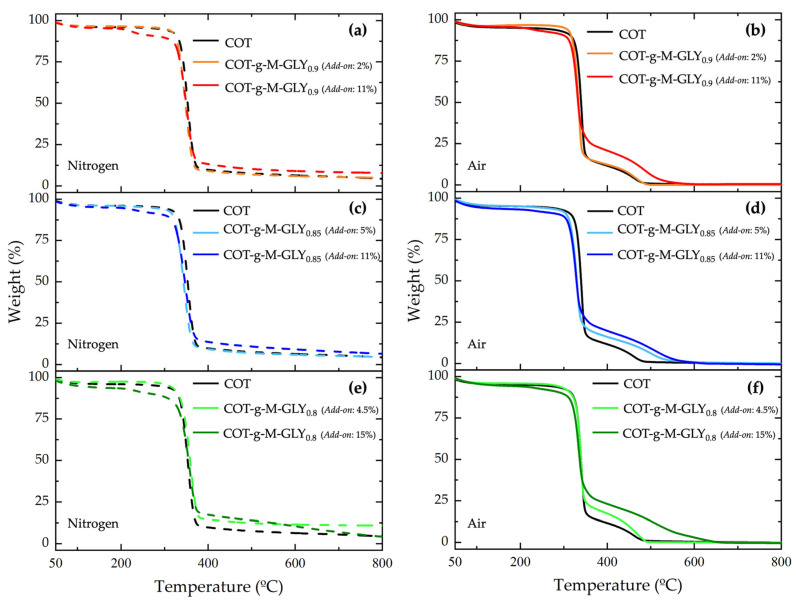
Comparison between the TG curves in nitrogen (**a**,**c**,**e**) and in air (**b**,**d**,**f**) of virgin cotton and COT-g-M-GLY samples.

The thermal and thermo-oxidative behavior of COT-g-M-GLY samples was evaluated through thermogravimetric analysis (TGA) under inert (nitrogen) and oxidative (air) atmospheres. The TG thermograms and corresponding thermal data are presented in Figure 4 and Table 4. In agreement with literature reports, untreated cotton exhibited a single-step decomposition, with maximum weight loss occurring at approximately 350 °C in nitrogen and 327 °C in air (Figure 4a,b) [17]. As previously reported for adsorbed polyamidoamine coatings [36], grafted M-GLY similarly promoted earlier thermal decomposition of cotton by slightly reducing the T_onset10%_ values in both nitrogen and air. It also enhanced thermal stability above 350 °C and improved thermo-oxidative stability in the 350–550 °C range (Table 4). However, although changes in the thermogravimetric profile became more pronounced with increasing M-GLY add-on, they remained less significant than those observed in COT/M-GLY samples with lower or comparable add-ons (Table 4 and Figure S6). For comparison, a COT/M-GLY sample with only 7% M-GLY add-on reduced T_onset10%_ to 270 °C in nitrogen and in air—T_onset10%_ was 247 °C in nitrogen and 256 °C in air when the add-on reached 14%—while T_onset10%_ decreased only to 270 °C in nitrogen and to 293 °C in air for a COT-g-M-GLY_0.8_ sample with an add-on as high as 15%. Analogously, COT/M-GLY with a 7% add-on exhibited an RMF of 40% in air at 350 °C (Figure S6), while COT-g-M-GLY_0.8_ with a 15% add-on exhibited an RMF of 32% in air at the same temperature. These findings suggest that COT-g-M-GLY samples do not exhibit the same intumescent behavior of COT/M-GLY samples with comparable add-on [23,36]. This effect is likely due to the physical constraints imposed by the crosslinked architecture of grafted M-GLY, which may alter its thermal and thermo-oxidative behavior.

### 3.4. Combustion Tests

**Figure 5:**
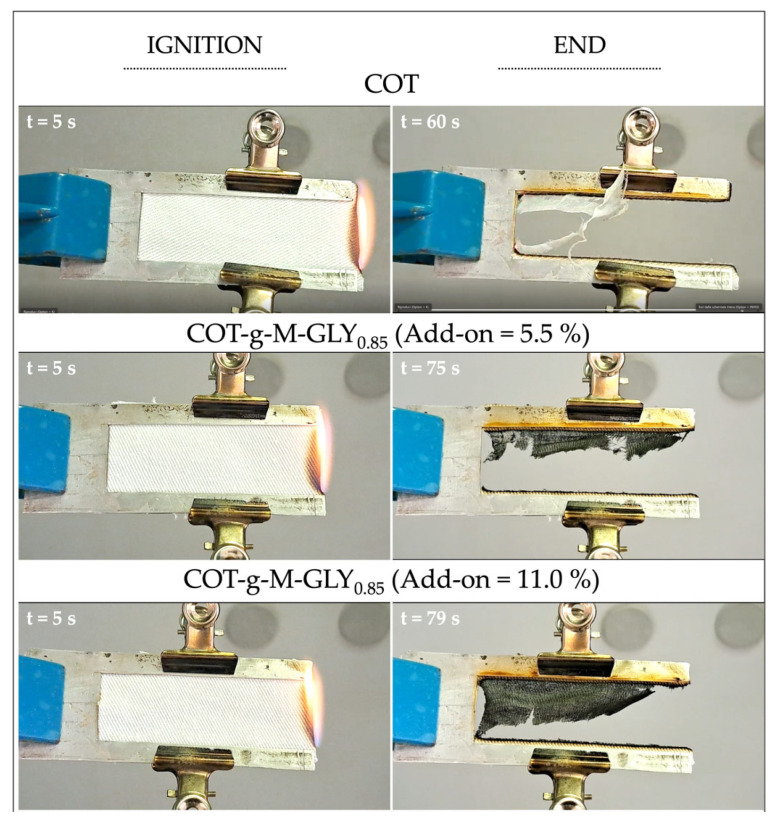
Snapshots of horizontal flame spread tests for virgin cotton (COT) and COT-g-M-GLY_0.85_.

The combustion behavior of COT-g-M-GLY samples was studied by horizontal flame spread tests (HFSTs). Combustion data are presented in Table 5, and representative images of combusted COT-g-M-GLY_0.85_ fabrics are shown in Figure 5. All collected data show that M-GLY coatings did not enable cotton to self-extinguish, even at the highest add-on levels. However, although the treated samples exhibited only modest RMF values—exceeding 5% for add-ons above 11.0%, compared to approximately 0.5% for virgin cotton—they significantly reduced the burning rate, which decreased by 10 to 30% as the add-on increased from 2% to 11% compared with virgin cotton. This behavior contrasts sharply with that of M-GLY-adsorbed cotton fabrics, where the M-GLY coating induced self-extinguishment at add-ons as low as 6–7%, while achieving RMF values >85% [31]. The lower flame-retardant efficacy of COT-g-M-GLY aligns with the limited thermo-oxidative stability observed in thermogravimetric experiments.

### 3.5. Accelerated Photoaging Tests

The photoaging resistance of COT-g-M-GLY_0.85_, chosen as a model compound, was evaluated through accelerated photoaging tests carried out on samples with a 10% M-GLY add-on, exposed to UVA and UVB irradiation for a total duration of 22 h. Experiments were conducted using untreated cotton as the reference material, along with COT/M-GLY samples with approximately 10% add-on obtained by impregnating cotton in an aqueous solution of M-GLY followed by drying. This add-on was chosen based on a previous study in which M-GLY proved to be effective in protecting cotton against UVA radiation [24].

**Figure 6:**
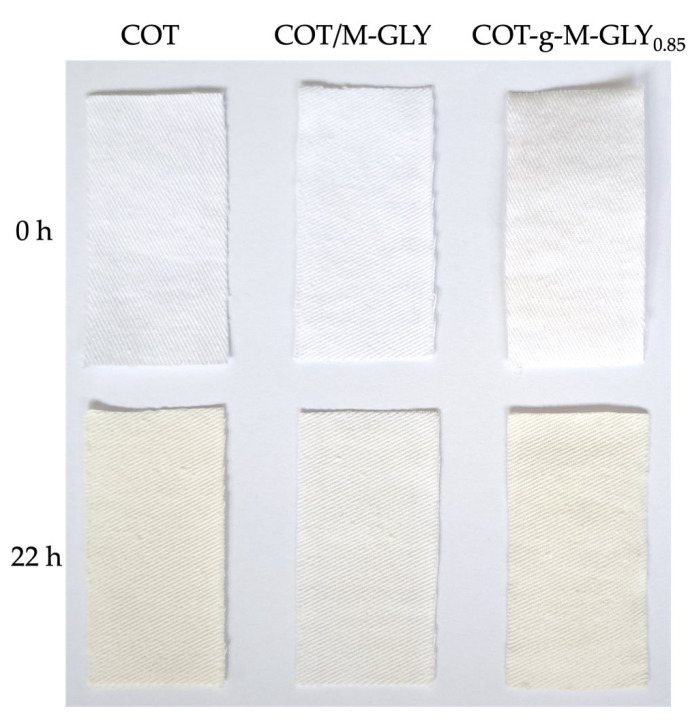
Digital pictures of virgin cotton (COT), COT/M-GLY, and COT-g-M-GLY_0.85_ strips before and after 22 h of photoaging. M-GLY add-on: 10 wt%.

Images of COT-g-M-GLY_0.85_, COT/M-GLY, and virgin cotton before and after the aging process are shown in Figure 6. It is worth noting that the initial color of COT-g-M-GLY_0.85_ appeared less bright than that of COT/M-GLY and untreated cotton, likely due to the removal of whitening additives during the chemical grafting process.

**Figure 7:**
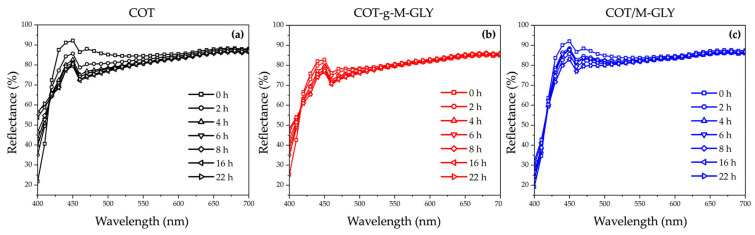
Reflectance spectra of virgin cotton (COT), M-GLY-adsorbed cotton (COT/M-GLY), and M-GLY-grafted cotton (COT-g-M-GLY_0.85_) at different photoaging stages. The M-GLY add-on of both COT/M-GLY and COT-g-M-GLY_0.85_ was about 10%. (**a**) COT; (**b**) COT-g-M-GLY; (**c**) COT/M-GLY.

Reflectance spectra obtained with virgin cotton, COT/M-GLY, and COT-g-M-GLY_0.85_ –both with an add-on of about 10%—after different irradiation times are shown in Figure 7. As observed in similar experiments [24], the maximum reflectance value of cotton, placed at 450 nm, significantly declined throughout the irradiation experiment. After 9 h of irradiation, the consecutively collected reflectance curves showed no significant variations, and after 22 h, the maximum value had decreased by a total of 16%. In contrast, COT/M-GLY showed a much slower decline in maximum reflectance, with a total decrease of the maximum value of 9% after 22 h. As visually observed, COT-g-M-GLY_0.85_ exhibited a slightly colored surface prior to photoaging, resulting in a lower initial maximum reflectance value at time zero. Nevertheless, the overall reduction in maximum reflectance after 22 h of irradiation was comparable to that of COT/M-GLY, that is, around 8%.

**Figure 8:**
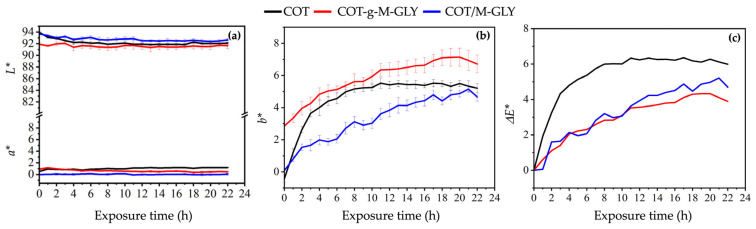
(**a**–**c**) Change of the color parameters of virgin cotton (COT), M-GLY-grafted cotton (COT-g-M-GLY_0.85_), and M-GLY-adsorbed cotton (COT/M-GLY) at different photoaging stages. Both COT/M-GLY and COT-g-M-GLY_0.85_ had an add-on of about 10%. L* = brightness; a*, b* = chromaticity coordinates; ΔE* = color change, as defined in Section 2.9.

Color changes in the photoaged virgin and treated cotton samples were more clearly characterized by analyzing the CIELAB color space parameters, namely L* (brightness), chromatic coordinates a* (green–red axis), b* (blue–yellow axis), and ΔE* (color change, defined according to Equation (3)). From a practical perspective, an ideal pure white has L* = 100, a* = 0 and b* = 0. The collected results are shown in Figure 8.

In all the cases considered, the L* values only slightly declined throughout the irradiation period, with a slightly more pronounced decline for virgin cotton (Figure 8a). A similar trend was observed for the a* coordinate, mirroring the behavior seen in the L* coordinate across all samples studied (Figure 8b). Notably, the b* coordinate, indicative of the yellow color component through its positive values, followed a different trend. In virgin cotton, the b* value increased sharply during the first 10 h of irradiation, indicating substantial and progressive yellowing (b_10h*_ = 5.8) (Figure 8c). However, beyond 10 h, the yellowing rate significantly decreased, although it never reached 0. Interestingly, the b* coordinate of both COT-g-M-GLY_0.85_ and COT/M-GLY increased at a steady and comparable rate throughout the entire duration of the experiment, with the only difference being the higher initial b* value of COT-g-M-GLY_0.85_. This rate of increase was significantly slower than that observed for untreated cotton during the first 10 h of irradiation. A clearer comparison emerges from the analysis of the ΔE* trend, which confirms the differing rates of color change between M-GLY-treated and untreated cotton fabrics, while also indicating that the overall color change of the COT-g-M-GLY_0.85_ and COT/M-GLY samples remained comparable over the full course of the experiment.

Consistent with the spectrophotometric analysis, FT-IR spectroscopy confirmed the early stages of photo-oxidation in COT and COT-g-M-GLY_0.85_ after 22 h of UVA–UVB exposure, evidenced by the emergence of a small peak at 1740 cm^−1^ in Figure S7, corresponding to carbonyl group absorption. This peak was not observed in the spectrum of the photoaged COT/M-GLY sample.

**Figure 9:**
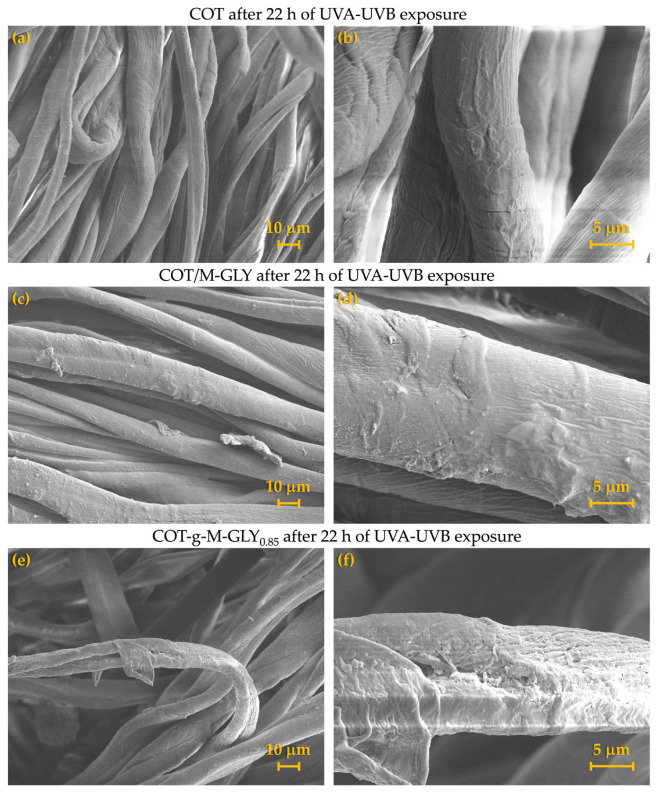
(**a**–**f**) FE-SEM micrographs of COT, COT/M-GLY, and COT-g-M-GLY_0.85_ (add-on: 10%) after 22 h of exposure to UVA–UVB radiation.

Following the accelerated photoaging tests, the morphology of COT, COT/M-GLY, and COT-g-M-GLY_0.85_ was examined by FE-SEM, revealing no significant morphological changes compared to the samples before exposure (see Figure 3 and Figure 9).

## 4. Conclusions

In this study, a water-based method was developed to graft durable polyamidoamine coatings onto cotton, aiming to enhance its physical and chemical properties. Glycine-based polyamidoamine oligomers (M-GLY), synthesized from N,N′-methylenebisacrylamide and glycine with excess acrylamide groups, were used as model compounds. These oligomers were applied to cotton at 80 °C in the presence of potassium persulfate, which generated radicals that initiated polymerization of terminal acrylamide groups, resulting in covalent grafting. The bifunctional nature of M-GLY led to the formation of a crosslinked polymer network, with add-ons ranging from 2–15%, depending primarily on oligomer concentration. Scanning electron microscopy confirmed the formation of a uniform, defect-free coating that penetrated the fiber structure. Thermal analysis (TG) showed that COT-g-M-GLY samples exhibited different decomposition behavior from cotton treated with unbound M-GLY (COT/M-GLY). Grafted samples showed less volatilization under oxidizing conditions, attributed to reduced intumescence due to crosslinking constraints. However, the crosslinked samples displayed lower flame retardancy, with longer flaming and afterglow durations and reduced char formation compared to their adsorbed counterparts. Photostability tests on COT-g-M-GLY_0.85_ (10% M-GLY add-on) revealed effective protection against UV-induced decomposition. Spectrophotometric data showed reduced yellowing rates, comparable to those achieved with adsorbed M-GLY. Infrared and electron microscopy confirmed the structural stability of the coating even after 22 h of UV exposure. Overall, the radical grafting process produced robust, homogeneous coatings that altered cotton’s properties in both expected and novel ways. Notably, grafting influenced thermal degradation differently than non-grafted systems, while photostabilization performance remained similar. These findings demonstrate the potential of covalently grafted PAAs to enhance cotton’s performance and represent a proof of concept in view of further studies using PAAs with varied structures.

## Figures and Tables

**Table 1:** Amounts of the reagents used in the synthesis of M-GLY oligomers.

	MBA		GLY		LiOH·H_2_O		H_2_O
	(g)	(mmol)	(g)	(mmol)	(g)	(mmol)	(mL)
M-GLY_0.9_	9.34	60	4.10	54	2.32	54	25
M-GLY_0.85_	9.34	60	3.87	51	2.19	51	25
M-GLY_0.8_	9.34	60	3.64	48	2.06	48	25

**Table 2:** Molecular masses of M-GLY oligomers ^(a)^.

Sample	Theoretical *r* ^(b)^ Value	Experimental *r* Value	*n* ^(c)^	${\bar{X}}_{n}$ ^(d)^	${\bar{M}}_{n}$ ^(e)^
M-GLY_0.9_	0.90	0.896	7	17	1990
M-GLY_0.85_	0.85	0.860	5	13	1530
M-GLY_0.8_	0.80	0.823	4	10	1300

^(a)^ Determined through ^1^H-NMR. ^(b)^ *r* = molar monomer ratio in the feed. ^(c)^ Number of internal repeat units. ^(d)^ Number-average degree of polymerization. ^(e)^ Number-average molecular mass. Experimental *r*, *n*, ${\bar{X}}_{n}$**,** and ${\bar{M}}_{n}$ were determined using Equations (S1), (S2), (S3), and (S4), respectively.

**Table 4:** Comparison among the thermal data of untreated cotton, COT/M-GLY, and COT-g-M-GLY fabrics.

Sample	Add-On (%)	T _onset10%_^(a)^ (°C)	T_max1_ ^(b)^ (°C)	T_max2_^(b)^ (°C)	RMF ^(c)^ (%)
Nitrogen					
COT	-	330	352	-	4.5
COT-g-M-GLY_0.9_	2	325	349	-	5
COT-g-M-GLY_0.9_	11	293	351	-	7
COT-g-M-GLY_0.85_	5	319	344	-	4.5
COT-g-M-GLY_0.85_	11	303	347	-	6.5
COT-g-M-GLY_0.8_	4.5	331	358	-	11
COT-g-M-GLY_0.8_	15	270	360	-	10
COT/M-GLY [47]	7	270	325	-	20
COT/M-GLY [42]	14	247	310	-	23
Air					
COT	-	310	327	523	0
COT-g-M-GLY_0.9_	2	315	333	460	0
COT-g-M-GLY_0.9_	11	303	330	486	0
COT-g-M-GLY_0.85_	5	306	328	498	0
COT-g-M-GLY_0.85_	11	291	330	510	0
COT-g-M-GLY_0.8_	4.5	315	339	470	0
COT-g-M-GLY_0.8_	15	293	334	509	0
COT/M-GLY	7	270	316	427	1
COT/M-GLY	14	256	304	433/481	0

^(a)^ Onset decomposition temperature at 10% weight loss. ^(b)^ From the dTG curve. ^(c)^ Residual mass fraction at 800 °C.

**Table 5:** Combustion data of COT-g-M-GLY fabrics from horizontal flame spread tests.

	Add-On ^(a)^ (%)	Combustion Time in the Presence of Flame (s) ^(b)^	Combustion Time in the Presence of Incandescence (s) ^(b)^	Burning Rate ^(c)^ (mm s^−1^)	RMF ^(d)^ (%)
Cotton	-	55	2	1.0	0.5
COT-g-M-GLY_0.9_	2	62	3	0.9	2
	12	73	2	0.8	6
COT-g-M-GLY_0.85_	5.5	66	5	0.8	2
	11	80	7	0.7	7
COT-g-M-GLY_0.8_	4.5	69	3	0.8	1.5
	15	77	4	0.7	7

^(a)^ Add-on ± 1%. ^(b)^ Average time ± 1 s. ^(c)^ Calculated with respect to the total combustion time. ^(d)^ Residual mass fraction.

## Data Availability

The original contributions presented in this study are included in the article/Supplementary Material. Further inquiries can be directed to the corresponding authors.

## Supplementary Materials

The following supporting information can be downloaded at: https://www.mdpi.com/article/10.3390/polym17121676/s1. Figure S1: ^1^H-NMR spectra of M-GLY_0.85_ (a) and M-GLY_0.8_ (b) oligomers; Figure S2: Digital pictures of a crosslinked hydrogel deriving from a M-GLY_0.85_ solution after 6 weeks of immersion in water (a), under static conditions (b), and under compression (c); Figure S3: FT-IR/ATR spectra of COT and COT-g-M-GLY samples; Figure S4: Spectral region of ^1^H-^13^C-CP-MAS spectra of COT-g-M-GLY_0.85_ (a) and pure cotton (b) showing the signals due to C4 carbon atoms in the β-D-glucopyranose repeat units; Figure S5: XRD spectra of untreated COT, COT-g-M-GLY_0.85_, and COT-g-M-GLY_0.8_; Figure S6: FT-IR/ATR spectra of COT, COT-g-M-GLY_0.85_, and COT/M-GLY before and after 22 h of exposure to UVA–UVB irradiation; Figure S7: FT-IR/ATR spectra of COT, COT-g-M-GLY0.85, and COT/M-GLY before and after 22 h of exposure to UVA–UVB irradiation; Table S1: Position of XRD signals and crystallographic parameters of COT, COT-g-M-GLY_0.85_, and COT-g-M-GLY_0.8_.

## References

[1] Delgado J.F., de la Osa O., Salvay A.G., Cavallo E., Cerrutti P., Foresti M.L., Peltzer M.A. (2021). Reinforcement of yeast biomass films with bacterial cellulose and rice husk cellulose nanofibers. J. Polym. Environ..

[2] Roy D., Semsarilar M., Guthrie J.T., Perrier S. (2009). Cellulose modification by polymer grafting: A review. Chem. Soc. Rev..

[3] Aziz T., Farid A., Haq F., Kiran M., Ullah A., Zhang K., Li C., Ghazanfar S., Sun H., Ullah R. (2022). A Review on the Modification of Cellulose and Its Applications. Polymers.

[4] Tosh B., Routray C.R. (2014). Grafting of Cellulose Based Materials: A Review. Chem. Sci. Rev. Lett..

[5] Mondal I.H., Uraki Y., Ubukata M., Itoyama K. (2008). Graft polymerization of vinyl monomers onto cotton fibres pretreated with amines. Cellulose.

[6] Takahashi A., Takahashi S. (1974). Graft Copolymerization onto Cellulose Derivatives. IV. Graft Copolymerization of Styrene and Styrene-Methyl Methacrylate onto Cellulosic Materials Containing Carbonyl and Carboxyl Groups. Polym. J..

[7] Wang L., Dong W., Xu Y. (2007). Synthesis and characterization of hydroxypropyl methylcellulose and ethyl acrylate graft copolymers. Carbohydr. Polym..

[8] Wei L., McDonald A.G. (2016). A Review on Grafting of Biofibers for Biocomposites. Materials.

[9] Kang H., Liu R., Huang Y. (2015). Graft modification of cellulose: Methods, properties and applications. Polymer.

[10] Daly W.H., Evenson T.S., Iacono S.T., Jones R.W. (2001). Recent developments in cellulose grafting chemistry utilizing Barton ester intermediates and nitroxide mediation. Macromol. Symp..

[11] Carlmark A., Malmstrom E. (2002). Atom Transfer Radical Polymerization from Cellulose Fibers at Ambient Temperature. J. Am. Chem. Soc..

[12] Rahaman H., Haque A., Rahman A., Rana M., Parvez M., Nur Alam S.M. (2022). Grafting of Cellulose and Microcrystalline Cellulose with Oligo(L-lactic acid) by Polycondensation Reaction. Reactions.

[13] Carlmark A., Larsson E., Malmström E. (2012). Grafting of cellulose by ring-opening polymerisation—A review. Eur. Polym. J..

[14] Shukla S.R., Athalye A.R. (1992). Ultraviolet-radiation induced graft-copolymerization of hydroxyethyl methacrylate onto cotton cellulose. J. Appl. Polym. Sci..

[15] Takacs E., Wojnarovits L., Borsa J., Papp J., Hargittai P., Korecz L. (2005). Modification of cotton-cellulose by preirradiation grafting. Nucl. Instrum. Methods B.

[16] Li J.F., Jiang W., Liu M.L. (2022). Durable phosphorus/nitrogen flame retardant for cotton fabric. Cellulose.

[17] Trovato V., Sfameni S., Ben Debabis R., Rando G., Rosace G., Malucelli G., Plutino M.R. (2023). How to Address Flame-Retardant Technology on Cotton Fabrics by Using Functional Inorganic Sol–Gel Precursors and Nanofillers: Flammability Insights, Research Advances, and Sustainability Challenges. Inorganics.

[18] Horrocks A.R. (1983). An introduction to the burning behaviour of cellulosic fibres. J. Soc. Dye. Colour..

[19] Qi P., Li Y., Yao Y., Sun J., Li L., Liu J., Gu X., Li H., Zhang S. (2023). Ultra washing durable flame retardant coating for cotton fabric by the covalent bonding and interface polymerization. Chem. Eng. J..

[20] Horrocks R.A. (2011). Flame retardant challenges for textiles and fibres: New chemistry versus innovatory solutions. Polym. Degrad. Stabil..

[21] Salmeia K.A., Gaan S., Malucelli G. (2017). Recent advances for flame retardancy of textiles based on phosphorous chemistry. Polymers.

[22] Hirschler M.M., Wilkie C.A., Morgan A.B. (2010). Fire Retardancy of Polymer Materials. Regulations, Codes, and Standards Relevant to Fire Issues in the United States.

[23] Chen Y., Li J., Liu L., Zhao N. (2012). Polybrominated diphenylethersfate in China: A review with an emphasis on environmental contamination levels, human exposure and regulation. J. Environ. Manag..

[24] Basak S., Wazedi Ali S. (2016). Sustainable flame retardancy of textiles using bio-macromolecules. Polym. Degrad. Stabil..

[25] Botti S., Di Lazzaro P., Flora F., Mezi L., Murra D. (2024). Raman spectral mapping reveal molecular changes in cellulose aging induced by ultraviolet and extreme ultraviolet radiation. Cellulose.

[26] Malesic J., Kolar J., Strlic M., Kocar D., Fromageot D., Lemaire J., Haillant O. (2005). Photo-induced degradation of cellulose. Polym. Degrad. Stabil..

[27] Merlin A., Fouassier J. (1980). Photochemical investigations into cellulosic materials I. Free radical generation in cellulose by photosensitized excitation. Angew. Makromol. Chem..

[28] Muasher M., Sain M. (2006). The efficacy of photostabilizers on the color change of wood filled plastic composites. Polym. Degrad. Stabil..

[29] Forte C., Alongi J., Beduini A., Borsacchi S., Calucci L., Carosio F., Ferruti P., Ranucci E. (2021). The Thermo-Oxidative Behavior of Cotton Coated with an Intumescent Flame Retardant Glycine-Derived Polyamidoamine: A Multi-Technique Study. Polymers.

[30] Alongi J., Treccani S., Comite V., Fermo P., Ferruti P., Ranucci E. (2024). Polyamidoamine-based photostabilizers for cotton fabrics. Polym. Degrad. Stabil..

[31] Ranucci E., Manfredi A. (2019). Polyamidoamines: Versatile bioactive polymers with potential for biotechnological applications. Chem. Afr..

[32] Argenziano M., Dianzani C., Ferrara B., Swaminathan S., Manfredi A., Ranucci E., Cavalli R., Ferruti P. (2017). Cyclodextrin-Based Nanohydrogels Containing Polyamidoamine Units: A New Dexamethasone Delivery System for Inflammatory Diseases. Gels.

[33] Cavalli R., Primo L., Sessa R., Chiaverina G., di Blasio L., Alongi J., Manfredi A., Ranucci E., Ferruti P. (2017). The AGMA1 Polyamidoamine Mediates the Efficient Delivery of SiRNA. J. Drug Target..

[34] Alongi J., Costantini A., Ferruti P., Ranucci E. (2022). Evaluation of the eco-compatibility of polyamidoamines by means of seed germination test. Polym. Degrad. Stabil..

[35] Ranucci E., Treccani S., Ferruti P., Alongi J. (2024). The Seed Germination Test as a Valuable Tool for the Short-Term Phytotoxicity Screening of Water-Soluble Polyamidoamines. Polymers.

[36] Treccani S., Ferruti P., Alongi J., Monti E., Zizioli D., Ranucci E. (2024). Ecotoxicity Assessment of α-Amino Acid-Derived Polyamidoamines Using Zebrafish as a Vertebrate Model. Polymers.

[37] Beduini A., Carosio F., Ferruti P., Ranucci E., Alongi J. (2021). Polyamidoamines Derived from Natural α-Amino Acids as Effective Flame Retardants for Cotton. Polymers.

[38] Beduini A., Carosio F., Ferruti P., Ranucci E., Alongi J. (2019). Sulfur-Based Copolymeric Polyamidoamines as Efficient Flame-Retardants for Cotton. Polymers.

[39] (2019). Colorimetry, Part 4: CIE 1976 L*a*b* Colour Space.

[40] GZ Schuessler 2024. https://zschuessler.github.io/DeltaE/learn/.

[41] Maggi F., Manfredi A., Carosio F., Maddalena L., Alongi J., Ferruti P., Ranucci E. (2022). Toughening Polyamidoamine Hydrogels through Covalent Grafting of Short Silk Fibers. Molecules.

[42] Manfredi A., Carosio F., Ferruti P., Ranucci E., Alongi J. (2018). Linear polyamidoamines as novel biocompatible phosphorus-free surface confined intumescent flame retardants for cotton fabrics. Polym. Degrad. Stabil..

[43] Newman R.H., Hemmingson J.A. (1990). Determination of the Degree of Cellulose Crystallinity in Wood by Carbon-13. Prog. Nucl. Magn. Reson. Spectrosc..

[44] French A.D. (2014). Idealized powder diffraction patterns for cellulose polymorphs. Cellulose.

[45] Zhao H., Kwak J.H., Zhang Z.C., Brown H.M., Arey B.W., Holladay J.E. (2007). Studying cellulose fiber structure by SEM, XRD, NMR and acid hydrolysis. Carbohydr. Polym..

[46] Salem K.S., Kasera N.K., Rahman M.A., Jameel H., Habibi Y., Eichhorn S.J., French A.D., Pal L., Lucia L.A. (2023). Comparison and assessment of methods for cellulose crystallinity determination. Chem. Soc. Rev..

[47] Beduini A., Ferruti P., Carosio F., Ranucci E., Alongi J. (2024). Synergism between α-amino acid-derived polyamidoamines and sodium montmorillonite for enhancing the flame retardancy of cotton fabrics. Polym. Degrad. Stabil..

