# Supplementary material for: Enhancing Cotton Fabrics Through Grafting of Glycine-Based Polyamidoamine
Source: Polymers (Basel). 2025 Jun 17;17(12):1676. doi: 10.3390/polym17121676 (PMC12197018; doi:10.3390/polym17121676)
Supplement: Supplementary file 1 [file polymers-17-01676-s001.zip › polymers-3695521-supplementary.pdf]

SUPPLEMENTARY MATERIALS

Enhancing cotton fabrics through grafting glycine-based polyamidoamine

Matteo Arioli, [1] Jenny Alongi [1], Claudia Forte[2], Silvia Pizzanelli[2] and Elisabetta Ranucci [1,*]

[1] Dipartimento di Chimica, Università degli Studi di Milano, via C. Golgi 19, 20133 Milano, Italy jenny.alongi@unimi.it (J.A.); matteo.arioli@unimi.it (M.A.); elisabetta.ranucci@unimi.it (E.R.)

[2] Institute of the Chemistry of OrganoMetallic Compounds (ICCOM), National Research Council, via G. Moruzzi 1, 56124 Pisa, Italy; claudia.forte@pi.iccom.cnr.it (C.F.); silvia.pizzanelli@pi.iccom.cnr.it (S.P.)

* Correspondence: elisabetta.ranucci@unimi.it (E.R.); Tel.: +29-02-50314132.

Pages S1-S9

Figure S1. ^{1}H-NMR spectra of M-GLY$_{0.85}$ (a) and M-GLY$_{0.8}$ (b) oligomers.

Figure S2. Digital pictures of a crosslinked hydrogel deriving from a M-GLY$_{0.85}$ solution after 6 weeks of immersion in water (a), under static conditions (b), and under compression (c).

Figure S3. FT-IR/ATR spectra of COT and COT-g-M-GLY samples.

Figure S4. Spectral region of ^{1}H-^{13}C CP-MAS spectra of COT-g-M-GLY$_{0.85}$ (a) and of pure cotton (b) showing the signals due to C4 carbon atoms in the β-D-glucopyranose repeat units.

Figure S5. XRD spectra of untreated COT, COT-g-M-GLY$_{0.85}$ and COT-g-M-GLY$_{0.8}$.

Figure S6. TG curves in nitrogen (a) and air (b) of COT, COT/M-GLY (add-ons: 7 and 14%) and COT-g-M-GLY$_{0.8}$ (add-on:15%).

Figure S7. FT-IR/ATR spectra of COT, COT-g-M-GLY$_{0.85}$, and COT/M-GLY at 0 h and after 22 h of exposure to UVA-UVB irradiation.

Table S1. Position of XRD signals and crystallographic parameters of COT, COT-g-M-GLY$_{0.85}$ and COT-g-M-GLY$_{0.8}$.

1. ^{1}*H-NMR characterization of PAA oligomers*

The chemical structure of M-GLY$_{0.85}$ and M-GLY$_{0.8}$ was assessed by ^{1}H-NMR, collecting spectra in D_2O at pH 4.0 and at 25°C using a Bruker Advance DPX-400 NMR spectrometer (Milan, Italy) operating at 400.13 MHz. Parameters: scan number 32, relaxation delay, *d1*, 10.0 s, receiver gain automatically measured and set by the instrument. Deuterium oxide (D_2O, >99.9%), and deuterium chloride solution (DCl, 35% in D_2O) were supplied by Sigma-Aldrich (Milan, Italy) and used as received.

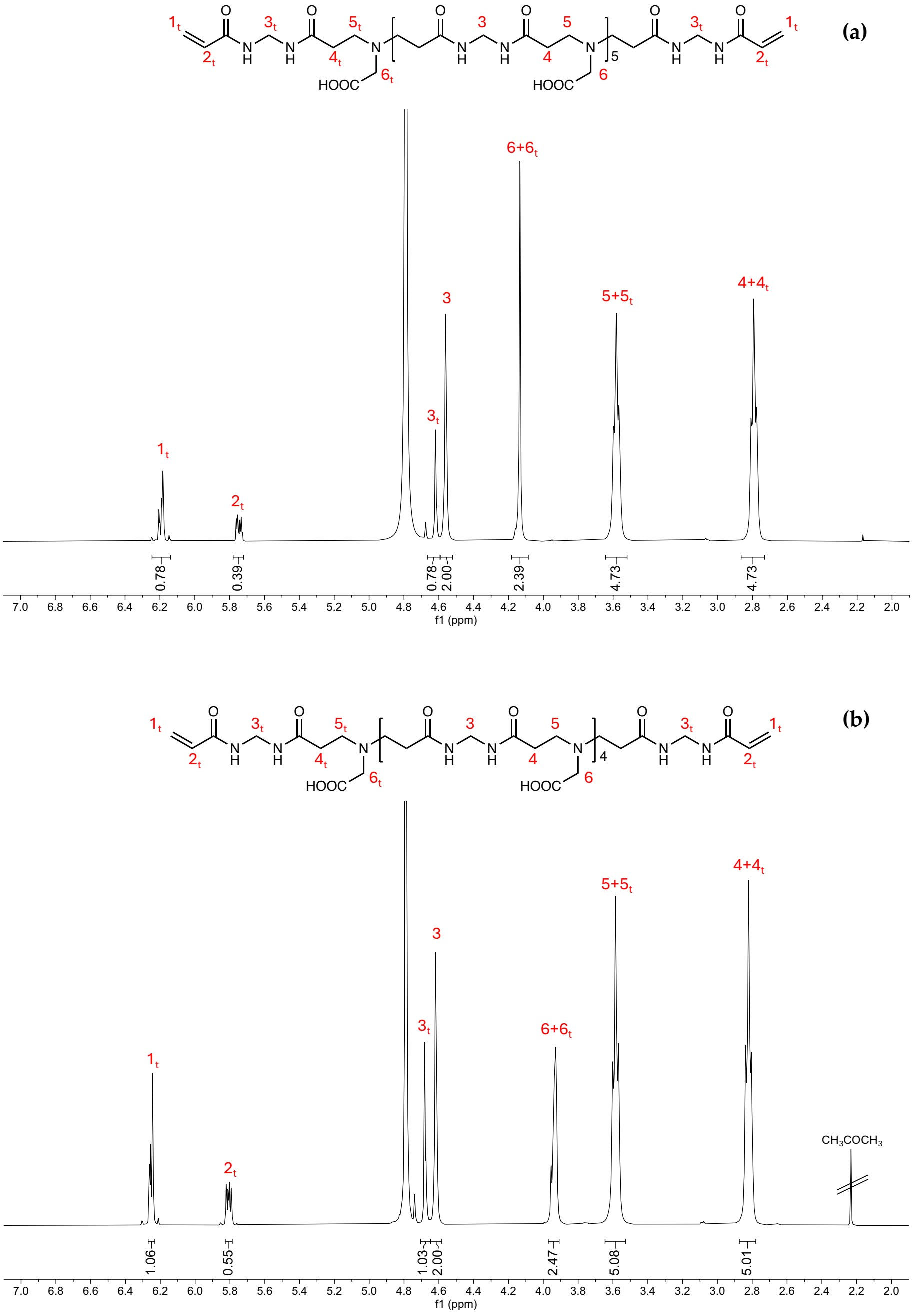

Figure S1. ^{1}H-NMR spectra of M-GLY$_{0.85}$ (a) and M-GLY$_{0.8}$ (b).

$\boldsymbol{r}$ (monomer ratio in the feed), $\boldsymbol{n}$ (number of the internal repeat units), $\overline{\boldsymbol{X}}_{\boldsymbol{n}}$ (number average polymerization degree) and $\overline{\boldsymbol{M}}_{\boldsymbol{n}}$ (number average molecular mass) were calculated through Equations S1-S4:

$$r = \frac{I_{H6} + I_{H6t}}{I_{H3} + I_{H3t}} \qquad \text{Eq. S1}$$

Where I_{H6} and I_{H3} represent the integrals of internal protons –and I_{H6t} and I_{H3t} those of the terminal protons– of the amine (glycine)-derived and acrylamide (MBA)-derived segments of the repeat units, respectively.

$$n = \frac{I_{H3}}{\frac{I_{H3t}}{2}} \qquad \text{Eq. S2}$$

$$\overline{X}_n = (n+1) \times 2 + 1 \qquad \text{Eq. S3}$$

$\overline{M}_n$was calculated following the Eq. 4:

$$\overline{M}_n = M_{AB} \times (n+1) + M_A \qquad \text{Eq. S4}$$

Where M_{AB} is the sum of the masses of monomers A and B, and M_A is the mass of monomer A.

2. *Synthesis of an M-GLY$_{0.85}$-based crosslinked hydrogel using the same conditions adopted in cotton grafting*

M-GLY$_{0.85}$ (0.8 g) and potassium persulfate (8 mg) were dissolved in ultrapure water (3.2 g) into a flat-bottomed plastic cylindrical container and allowed to react at 85 °C for 3 h. A transparent hydrogel was obtained that was carefully removed from the container, weighed, and placed in Milli-q water for 24 h. After this time, the swollen hydrogel was weighed, showing an 11.6 wt% mass increase.

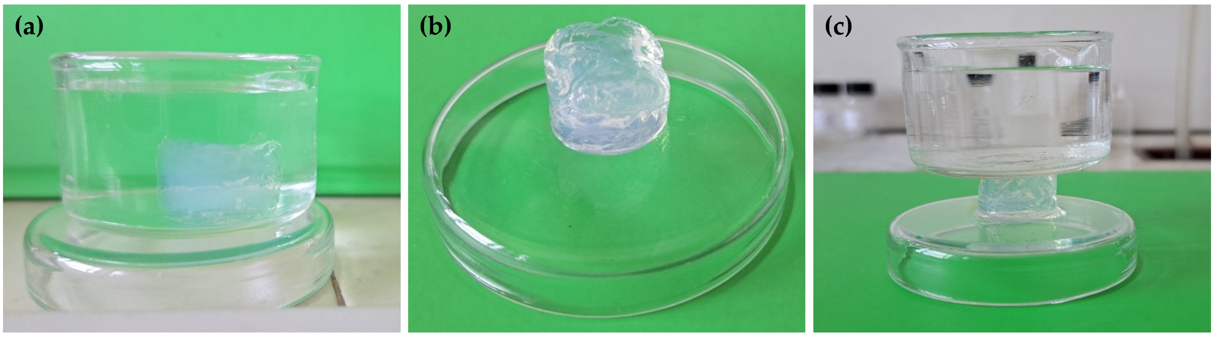

Figure S2. Digital pictures of a crosslinked hydrogel deriving from a M-GLY$_{0.85}$ solution after 6 weeks of immersion in water (a), under static conditions (b), and under compression (c).

3. FT-IR/ATR characterization of M-GLY-grafted cotton fabrics

COT (S3a-c), COT-g-M-GLY$_{0.9}$ (S3a), COT-g-M-GLY$_{0.85}$ (S3b) and COT-g-M-GLY$_{0.8}$ (S3c) were analyzed by attenuated total reflectance (ATR) Fourier-transform infrared spectroscopy (FT-IR). FT-IR/ATR spectra were recorded at room temperature, in the 4000 - 600 cm^{-1} wavenumber range, with 64 scans and 8 cm^{-1} resolution, using a Jasco FT-IR/FIR spectrophotometer (Milan, Italy), equipped with a diamond crystal.

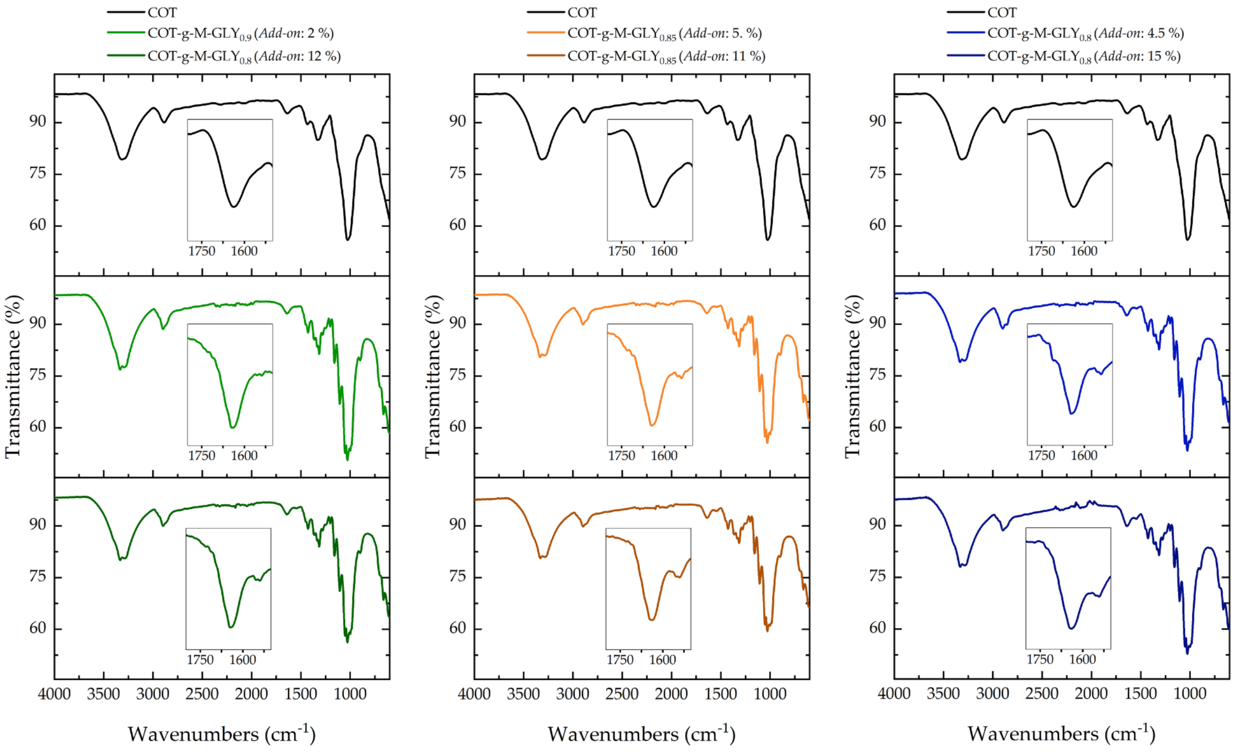

(a) **(b)** **(c)**

Figure S3. FT-IR/ATR spectra of COT and COT-g-M-GLY samples.

4. *Solid state NMR characterization of M-GLY-grafted cotton fabrics*

Solid-state NMR (SSNMR) spectra were acquired using a Bruker AVANCE NEO NMR spectrometer operating at Larmor frequencies of 500.13 MHz for ^{1}H and 125.77 MHz for ^{13}C nuclei. The instrument was equipped with a double-channel (H/F–X) 4 mm CP-MAS probe. The ^{1}H–^{13}C cross-polarization (CP) experiments were performed with high-power ^{1}H decoupling, using a contact time of 1 ms, a recycle delay of 2 s, and acquiring 4000 scans. The 90° pulse durations were 4.3 μs for ^{1}H and 4.2 μs for ^{13}C. Experiments were conducted under magic-angle spinning (MAS) at a spinning rate of 15 kHz and with high-power ^{1}H decoupling. The ^{13}C chemical shift scale was referenced to the external adamantane signal at 38.48 ppm.

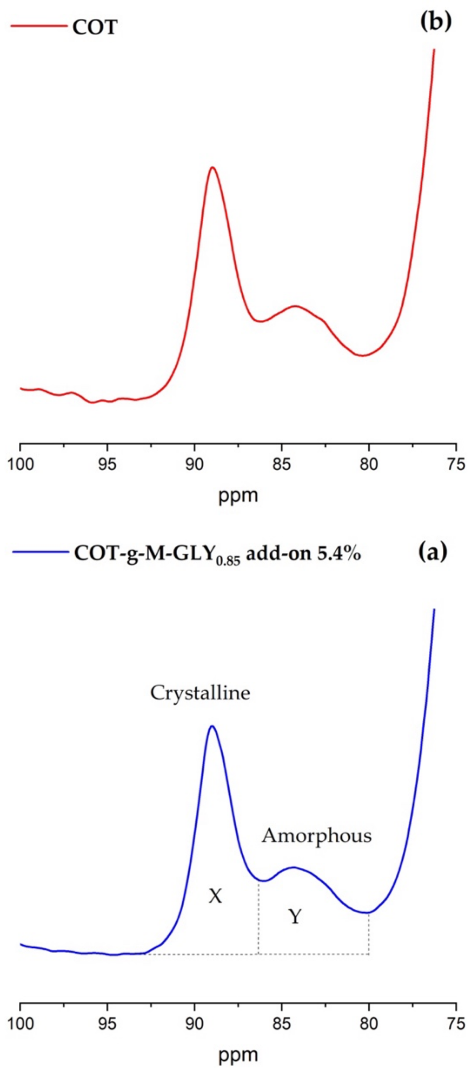

Figure S4. Spectral region of ^{1}H-^{13}C CP-MAS spectra of COT-g-M-GLY$_{0.85}$ (a) and of pure cotton (b) showing the signals due to C4 carbon atoms in β-D-glucopyranose repeat units.

5. *X-ray diffraction of M-GLY-grafted cotton fabrics*

The X-ray diffraction (XRD) spectra of virgin cotton and COT-g-M-GLY samples were recorded using a Miniflex 600 diffractometer with Cu $K_{\alpha 1}$ radiation at 1.5405 Å, at 40 kV voltage and 15 mA current (Rigaku Europe SE, Germany).

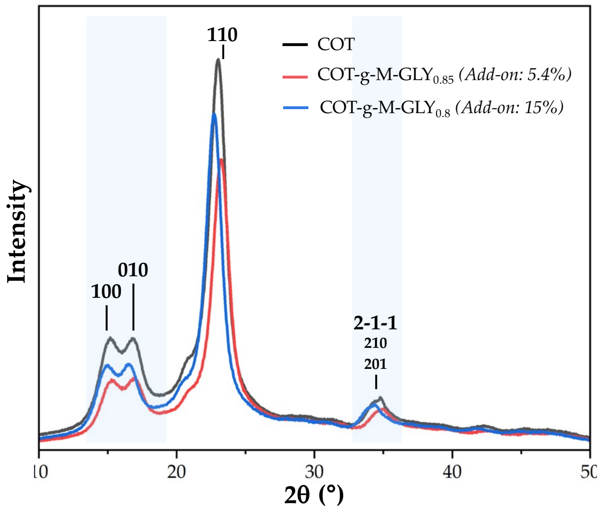

Figure S5. XRD spectra of untreated cotton (COT) and two examples of COT-g-M-GLY samples at different add-ons. COT-g-M-$GLY_{0.85}$, add-on: 5.4%; COT-g-M-$GLY_{0.8}$, add-on: 15%.

Table S1. Position of XRD signals and crystallographic parameters of COT, COT-g-M-$GLY_{0.85}$ and COT-g-M-$GLY_{0.8}$.

**Sample**	**2θ**			**d (nm)**			**Crystallinity index (%)**
**Assignment**	100	010	110	100	010	110	-
**COT**	15.21	16.76	22.98	0.294	0.267	0.197	64
**COT-g-M-$GLY_{0.85}$**	15.28	16.98	23.24	0.292	0.264	0.195	65
**COT-g-M-$GLY_{0.8}$**	14.96	16.48	22.76	0.298	0.272	0.199	66

6. Thermogravimetric analysis

The thermal stability of virgin cotton and COT-g-M-GLY samples was assessed by thermogravimetric analysis in nitrogen and air from 50 to 800 °C range, at 10 °C min^{-1} heating rate, with a 50 mL min^{-1} gas flow, using a TGA 2 Star System (Mettler-Toledo, Milan, Italy).

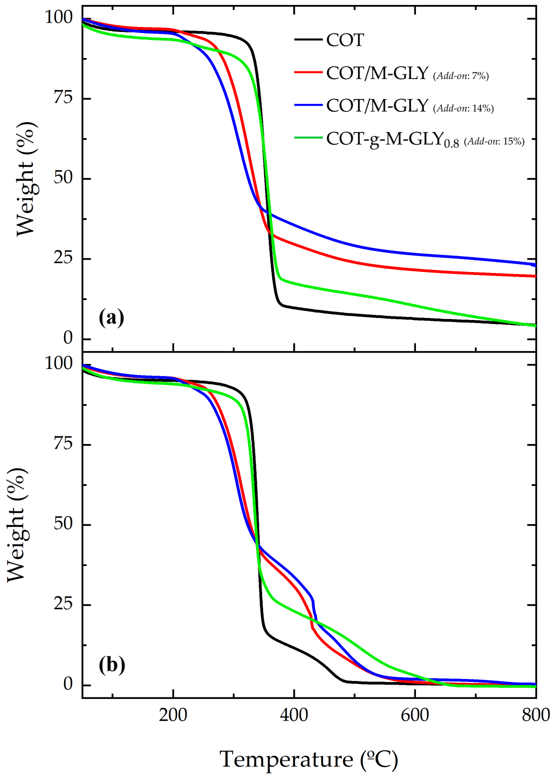

Figure S6. TG curves in nitrogen (a) and air (b) of COT, COT/M-GLY (add-ons: 7 and 14%) and COT-g-M-GLY$_{0.8}$ (add-on:15%).

7. FT-IR/ATR characterization of photoaged M-GLY-based cotton fabrics

COT (a), COT-g-M-GLY$_{0.85}$ (b) and COT/M-GLY (c) at 0 h and after 22 h of exposure to UVA-UVB irradiation were analyzed by FT-IR/ATR. Spectra were recorded at room temperature, in the 4000 - 600 cm^{-1} wavenumber range, with 64 scans and 8 cm^{-1} resolution, using a Jasco FT-IR/FIR spectrophotometer (Milan, Italy), equipped with a diamond crystal.

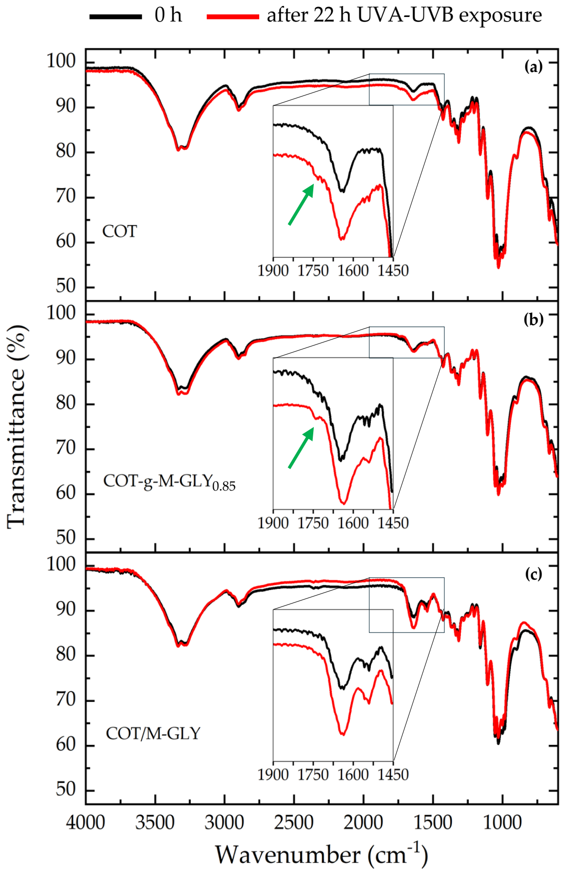

Figure S7. FT-IR/ATR spectra of COT, COT-g-M-GLY$_{0.85}$, and COT/M-GLY at 0 h and after 22 h of exposure to UVA-UVB irradiation.
